# *MMSA-1* is regulated by *Wnt/TCF4* and involved in multiple myeloma progression and invasion via *RAS/RAF* signaling pathway

**DOI:** 10.1007/s00277-026-06740-8

**Published:** 2026-01-15

**Authors:** Shan Meng, Hailing Liu, Liufang Gu, Jin Wang, Jianli Wang, Wanhong Zhao

**Affiliations:** Department of Hematology, the Second Affiliated Hospital of Xi’an Jiaotong University, West Five Road, NO. 157, Xi’an, Shaanxi Province 710004 P.R. China

**Keywords:** Multiple myeloma, MMSA-1, Β-catenin, TCF4, RAS

## Abstract

As a novel multiple myeloma (MM) specific antigen, rare is known about the underlying molecular mechanism of *MMSA-1* gene in the progression of myeloma. T*ranscription factor 4 (TCF4)* and *MMSA-1* over/down expressed stable U266 cell lines was constructed using lentivirus transfection technique. *TCF4*’s impact on *MMSA-1* expression was explored. Overexpressed of MMSA-1’s interaction with RAS protein and its downstream signaling pathways was investigated. Moreover, the interaction changes between overexpressed MMSA-1 protein and bone marrow microenvironment were also detected by examing adhesion molecules and angiogenesis promoting factors using Western Blot. We successfully constructed *transcription factor 4 (TCF4)* and *MMSA-1* over/down expressed stable U266 cell lines (termed ^*TCF4−/+*^U266 and ^*MMSA−1−/+*^U266). Our result showed that *TCF4* could bind with *MMSA-1* promoter sequence, greatly up regulate its promotor activity and then improve *MMSA-1* expression. Overexpressed *MMSA-1* also made a series of changes in U266 cells, including promoting RAS protein expression in cytoplasm, enhancing the interaction between *MMSA-1* and *RAS*, which resulted in hyperactivation of RAS and its downstream signaling pathways including RAF/MEK/ERK and RAF/PI3K/AKT, improving U266 cells’ clonogenicity capacity, changing apoptosis related proteins, reducing the interaction between myeloma cell and bone marrow microenvironment by reducing adhesion molecules expression including HIF-1α, E-cadherin, CXCR4 expression and elevating angiogenesis promoting factors including VEGF, Ang-2 and reducing Ang-1 at the same time. These results suggested *MMSA-1* was over expressed in MM cells being regulated by *Wnt/β-catenin/TCF4* signaling pathway, which resulted in hyperactivation of downstream RAS/RAF signaling pathway and eventually promote myeloma cells survival and invasion.

**Supplementary Information:**

The online version contains supplementary material available at 10.1007/s00277-026-06740-8.

## Introduction

Despite the great treatment improvement we have achieved in multiple myeloma (MM) in recent decades, it remains an incurable hematological malignancy and most patients eventually relapse and die after a plateau period [1]. The most fundamental problem is that the underlying complex pathological mechanism remains unclear [2]. More understanding about its pathogenesis and targeted therapy largely depends on discovery of novel myeloma associated genes.

*Multiple Myeloma Specific Antigen-1* (*MMSA-1)* gene, also known as a novel splicing variant of *ZDHHC9* gene, has been proved to be a multiple myeloma specific antigen which is relatively specifically overexpressed on myeloma cells and play important roles in myeloma cells proliferation, apoptosis and migration, as reported previously [3–5]. While rare is known about its overexpression mechanism as well as its molecular pathogenetic roles in myeloma patients, which need to be further investigated to provide some treatment clue for us.

On the other hand, *ZDHHC9*, a known gene closely related with RAS protein has been found to be over expressed in kinds of human malignancies such as breast cancer and colon cancer [6, 7]. It has been proved that in breast cancer cell line, overexpressed *ZDHHC9* could change RAS protein palmitoylation level and cellular localization, which activated RAS protein and downstream signaling pathways, and eventually result in losing control of cancer cells proliferation, migration and apoptosis. While as a novel splicing variant of *ZDHHC9*, whether *MMSA-1* plays a similar role in MM progression remains unclear, which also need to be further explored.

Herein, in this study, we performed a series of investigations in the aim of discovering regulation mechanism of *MMSA-1* gene in myeloma cells as well as its molecular pathogenetic functions in driving myeloma progression in order to provide more understanding about the biological function of *MMSA-1* gene and at the same time to find treatment clue in the near future.

## Materials and methods

### Cell lines

Human multiple myeloma cell line U266 and 293 T were purchased from Wuhan Procell Life Science&Technology Co., Ltd, both of which have been identified by Short Tandem Repeat (STR). Cells were cultured in RPMI1640 (for U266) or DMEM (for 293 T) medium added with 10% fetal bovine serum, 100 mg/ml streptomycin and 100 IU/ml penicillin in 5% CO_2_. Lentivirus expression vector (GV570, GV569, GV656 and GV112) as well as *transcription factor 4* (*TCF4)* and *MMSA-1* targeted RNAi sequences were designed and synthesized by Shanghai Genechem Co., LTD. Quantitative reverse transcription polymerase chain reaction (qRT-PCR) kit was purchased from (TianGen, Beijing, China). Dual-Luciferase^®^ Report Assay Systems was purchased from Promega (Wisconsin, US). TCF4 antibody for Western Blot was purchased from Cell signaling technology (Massachusetts, US), MMSA-1 antibodies for immunohistochemistry and Western Blot were purchased from (Abcam, Cambridge, UK), and glyceraldehyde-phosphate dehydrogenase (GAPDH) antibody was purchased from Goodhere Biotech Co., LTD (Hangzhou, Zhejiang, China).

### ^*TCF−/+*^U266 and ^*MMSA−1−/+*^U266 stable cell lines construction

U266 cells were cultured in RPMI1640 added with 10% fetal bovine serum and 100 mg/ml streptomycin and 100 IU/ml penicillin in 5% CO_2_. Then ^*TCF4−/+*^U266 cells and ^*MMSA−1−/+*^U266 cells were constructed as previously reported. In brief, GV569 and GV570 vector were used for *TCF4* over/down expression cell lines construction, respectively and GV656 and GV112 vectors were used for *MMSA-1* over/down expression cell lines construction, respectively, with protocols described as manufacturer’s instructions. An empty vector (EV) was used as negative control. Transfection was performed using Lipofectamine 2000 Transfection Reagent (Invitrogen, Carlsbad, CA) according to the manufacturer’s instructions. siRNAs targeting human *TCF4* (5’-GTCCGAGAAAGGAATCTGAAT-3’) and *MMSA-1* (5’-GTTACACATGCAAGATCTT-3’) respectively were designed and synthesized and cloned into the vectors by the GeneChem Corporation (Shanghai, China). Nontargeting plasmids (termed as ^*TCF4−/+*^U266-NC and ^*MMSA−1−/+*^U266-NC) were used as negative controls. After that, *TCF4* and *MMSA-1* expression level were examined using qRT-PCR to verify the successful construction of the cell lines.

### qRT-PCR and Western Blot to identify *MMSA-1* expression in ^*TCF4−/+*^U266 cell lines

qRT-PCR was performed to examine *MMSA-1* mRNA expression level in ^*TCF4−/+*^U266 cell lines using a PTC-1000 programmable thermal controller (MJ Research, Waltham, MA, US) with a PrimeScript ^®^RT-PCR kit (TianGen Bio. Inc., Shiga, Japan) according to the manufacturer’s instructions. In brief, the total RNA of the constructed U266 cells was isolated with an RNeasy Mini kit (QianGen, Valencia, CA). The TaqMan Reverse Transcription Kit (UE, Suzhou, China) and a Gene Amp polymerase chain reaction (PCR) System was performed to generate cDNA. Then PCR was performed with the qPCR superMIX (TianGen Bio. Inc., Shiga, Japan). The results were analyzed by the 2^−ΔΔCT^ method. The reduced GAPDH expression was used for gene expression normalization. The genes primers sequences were as follows: *TCF4* forward primer: 5’-AATACAAAGTAAAACAGAAAGGGG-3’, reverse primer:5’-TGATGGAGCATAGACTGAAGAT-3’; *MMSA-1* forward primer: 5’-TGAGAAAGAAGGTGACACGGAA-3’, reverse primer: 5’-GAAGAGCATGGCAGCAAATACA-3’; *GAPDH* forward primer: 5’-TCAAGAAGGTGGTGAAGCAGG-3’; reverse primer: 5’-TCAAAGGTGGAGGAGTGGGT-3’.

As for Western Blot assay, in brief, after the constructed ^*TCF4−/+*^U266 cells were collected and prepared, total protein was extracted, examined and calculated, and then subjected to sodium dodecyl sulfate-polyacrylamide gel electrophoresis analysis. After transferring the protein onto polyvinylidene fluoride membrane and subsequent blocking, the membranes were immunoblotted with rabbit anti-human TCF4 primary antibody (dilution ratio 1:1000, Cell signaling, Massachusetts, US), rabbit anti-human MMSA-1 primary antibody (dilution ratio 1:1000, Abcam, Cambridge, UK) overnight at 4 °C, followed by incubation with horseradish peroxidase (HRP)-conjugated goat anti-rabbit IgG secondary antibody (dilution ratio 1:50000, Biostech, Wuhan, China) for 2 h at room temperature. This was followed by detection of the HRP signal by enhanced chemiluminescence (ApplyGen, Beijing, China). BandScan software was applied to analyze the results.

### Chromatin immunoprecipitation (ChIP)

ChIP assays were performed with protocols as previously described [8, 9]. Briefly, after cells (including U266 cells, ^*TCF4−*^U266 and ^*TCF4+*^U266 as well as ^*TCF4−*^U266-NC and ^*TCF4+*^U266-NC which used as negative controls) were harvested, 1% formaldehyde was added and incubated at room temperature for 10 mins to cross-link protein and chromatin DNA. Then the cross-link reaction was stopped by adding glycine solution (1.25 mol/L). The complexes were then washed, and sonicated, precleared, and incubated with primary antibodies against human TCF4 or IgG (as a negative control). Complexes were washed with low- and high-salt buffers to reverse cross linking between targeted DNA and the bound protein. After that, the liberated DNA was extracted and precipitated. The precipitated DNA fragments were analyzed utilizing PCR with the following *MMSA-1*promoter primers: Forward primer: 5’-AGCCCCTTTCCTTTTTTG-3, Reverse primer: 5’-CACGTTGCCTGCTATTCC-3’. Non-immunoprecipitated chromatin fragments were used as an input control.

### Dual-luciferase report assay

*TCF4* over expression plasmid (termed as TCF4 plasmid) as well as negative control (termed as TCF4-NC) were successfully constructed as described above. The promoter sequence of *MMSA-1* was obtained on from Genebank website (https://www.ncbi.nlm.nih.gov/gene/51114). The upper stream of coding sequences with 1942 bp was thought to be the promoter sequence of *MMSA-1*, with sequence details shown in supplementary data 1 (https://www.ncbi.nlm.nih.gov/nuccore/NC_000023.11?report=genbank&from=129803288&to=129843886&strand=true). Then the fragment of pUC57-*MMSA-1* promoter was sub-cloned into pGL3-basic vector (Youbio, Changsha, P.R. China) to obtain pGL3-basic-*MMSA-1* promoter reporter plasmid. The constructed reporters were termed as *MMSA-1*-wt-NC and *MMSA-1*-wt. 293 T cells were co-transfected with the abovementioned reporter plasmids. The luciferase intensities were determined using the dual-luciferase reporter assay kit (Promega). Firefly luciferase intensity was normalized to Renilla luciferase activity. Dual-luciferase reporter assay was conducted three times with three biological repetitions each time.

### Immunofluorescence assay

Double fluorescent labeling technique was used to explore MMSA-1 and RAS proteins cellular localization. Constructed ^*MMSA-1-*^U266, ^*MMSA-1+*^U266 as well as control cells (U266, ^*MMSA-1-*^U266-NC and ^*MMSA-1+*^U266-NC) were cultured as described previously. Collected cells were seeded in cell culture plate preseeded with cell-attaching slides, 4% paraformaldehyde was added to fix cells for 15 mins followed by washing with phosphate buffered solution (PBS) for 3 times. Goat serum was added and incubated for 30 mins at room temperature. Then rabbit anti-human MMSA-1 polyclonal antibody (dilution ratio: 1:100, Abcam) were added and incubated for night at 4℃. Then Cy3 labeled goat anti-rabbit monoclonal antibody (dilution ratio: 1:100, Boster Biological Technology, Wuhan) was added, incubated at 37℃ for 1 h and washed. Then mouse anti-human RAS monoclonal antibody (dilution ratio: 1:100, Santa Crus) as well as Fluorescein Isothiocyanate (FITC)-labeled goat anti-mouse monoclonal antibody (dilution ratio: 1:100, Boster Biological Technology, Wuhan) was added with protocols described above. After that, 4’,6-Diamidino-2’-phenylindole (Dapi) was used to stain cell nucleus. Fluorescence microscopy was applied to take photos.

### Co-immunoprecipitation (Co-IP)

Co-IP was performed to examine the interaction of MMSA-1 and RAS proteins. In brief, different U266 cells (U266, ^*MMSA−1−*/*+*^U266 and ^*MMSA−1−/+*^U266-NC) were cultured and collected, washed and IP lysis solution was added to obtain the targeted protein. Agarose Protein A/G (Beyotime, Jiangsu, China) was washed with cold PBS and diluted to proper concentration (50%). Then Agarose Protein A/G was added to the prepared protein. After incubating the complex at 4℃ for 2 h, it was centrifuged at 3000 r/min for 5 min at 4℃ to remove Protein A/G. Co-IP mouse anti-human RAS monoclonal antibody (Santa Cruz Biotechnology, CA, USA) was used to form bead-antibody complexes. After incubated and washed, the sediment in the tube was collected to perform Western Blot analysis.

### Western Blot for RAS downstream signaling pathway related proteins

Western Blot analysis was applied for examination of RAS downstream signaling pathway related proteins’ expression with protocols described above. Antibodies were used as follows: rabbit anti-human MMSA-1 polyclonal antibody was purchased from Abcam (dilution ratio: 1:1000), rabbit anti-human p-pI3k, p-RAF, p-MEK, p-ERK antibodies were purchased from Cell Signaling Technology (dilution ratio: 1:1000) and rabbit anti-human p-AKT antibody were purchased from Affinity Biosciences (dilution ratio: 1:1000). Mouse anti-human RAS monoclonal antibodies were purchased from Santa Crus (dilution ratio: 1:200). HRP-labeled goat anti-rabbit/mouse antibodies were purchased from Boster biological technology (dilution ratio: 1:10000). Other protocols were performed as previously described. BandScan software was used to analyze the result.

### Double layer soft-agar clonogenicity capacity

To assess the clonogenecity capacity of different U266 cells, double layer soft-agar clonogenicity capacity assay was performed. In brief, stable cell line ^*MMSA−1*−/*+*^U266 cells and three control cells were cultured in RPMI 1640 added with 10% fetal bovine serum in 5% CO_2_. Cells were harvested, and resuspended into 5 × 10^3^/ml. The bottom and top agar concentration were 1.2% and 0.7%, respectively. After double layer soft-agar was prepared, cells were plated into 6-well plates with an initial density of 500 cells per well. After culturing for 2 weeks, generated colonies were immobilized by cooled alcohol, stained with 0.1% crystal violet at indoor temperature and counted. Colony formation rate indicates the percentage of the number of colonies among the number of seeded cells.

### Transwell assay

The migration and invasion of different U266 cells were analyzed using a transwell system (Costar-Corning, USA) in the absence or presence of Matrigel pre-coating (BD Biosciences, USA). Five kinds of U266 cells (U266, ^*MMSA1−1−/+*^U266-NC and ^*MMSA−1−/+*^U266) were cultured in RPMI 1640 + 10%FBS + 1% penicillin-streptomycin solution. Different cells were harvested, collected and washed by PBS, and then serum-free medium were added. 24 well plate pre-added with 800 µl RPMI 1640 medium with 10% FBS and 1% penicillin-streptomycin were put in transwell chamber. 1 h later, 1 × 10^5^ cells (200 µl) were seeded in the upper chamber. After culturing at 37℃, 5% CO2 for 24 h, transwell chamber were took out and cells were collected and transferred to 96 well culture plate. 10 µl CCK-8 were added and OD 450 were then examined.

### Apoptosis related molecule expression and mitochondrial apoptosis

Cell apoptosis inducer CCCP (5 µM) was used to amplify the difference between different cell lines. After 24 h pretreated with CCCP, Western Blot was performed to detect apoptosis related molecules including B cell lymphoma (BCL)−2, B cell lymphoma-extra large (BCL-XL) as well as BCL-2 associated X protein (BAX), Caspase-3, Caspase-8 and Caspase-9 levels with protocols described previously. Rabbit anti-human BCL-2, BAX, Caspase 3 and Caspase 9 polyclonal antibodies were purchased from Proteintech Group, Wuhan, rabbit anti-human BCL-XL polyclonal antibody was purchased from Affinity Bioscience, Shanghai and rabbit anti-human Caspase 8 polyclonal antibody was purchased from Bioworld Technology, Minnesota. All the antibodies mentioned above were diluted as the manufacturer’s suggestions.

To investigate whether MMSA-1 expression level have effect on myeloma cells’ mitochondrial apoptosis, mitochondrial apoptosis assay kit (Beyotime, Nanjing, China) was applied. After pretreated with apoptosis inducer CCCP (5 µM) for 24 h, different U266 cells were collected and subsequent to mitochondrial membrane potential (MMP) detection protocols followed by the manufacturer’s instruction.

### Adhesion molecules and angiogenesis promoting factors expression

qRT-PCR and Western Blot was performed to examine adhesion molecules expression including HIF-1α, E- cadherin and CXCR4 in different U266 cells, while Enzyme-Linked Immunosorbent Assay (ELISA) was applied to examine angiogenesis promoting factors including vascular endothelial growth factor (VEGF), Ang-1 and Ang-2 expressions with protocols described as the manufacturer’s instructions.

### Statistical analysis

All the experimental data were repeated three times. Statistical differences between experimental groups were analyzed using SPSS 18.0 or Graphpad Prism 5 software using the student’s *t* test or single factor ANOVA to assess the *P* values in correlations. *P <* 0.05 was considered statistically significant.

## Results

### ^*TCF4−/+*^U266 and ^*MMSA−1−/+*^U266 stable cell lines construction, identification and *TCF4’s* regulation on *MMSA-1* expression both in RNA and protein level

**Fig. 1:**
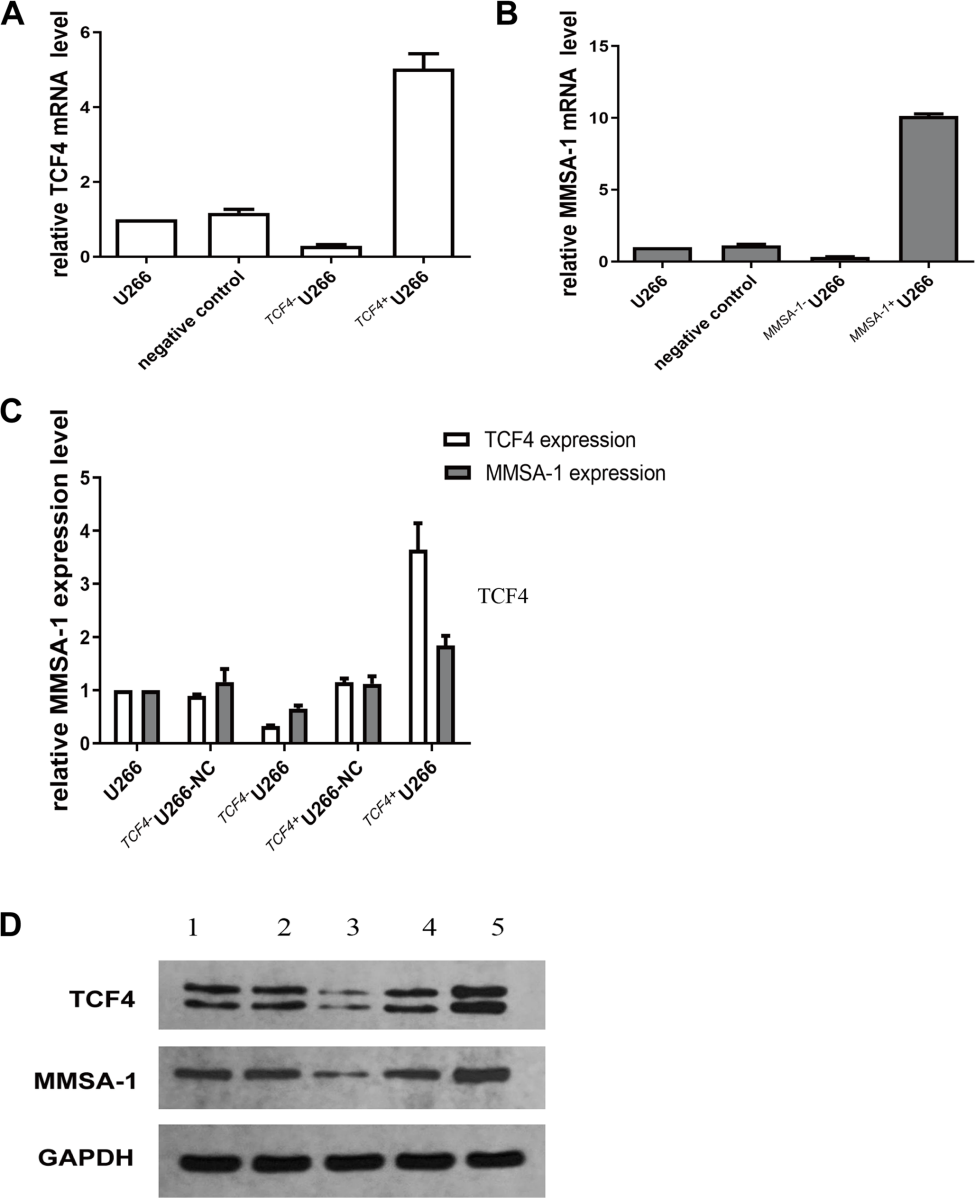
Identification of constructed ^***TCF4−/+***^U266 and ^***MMSA−1−/+***^U266 stable cell lines and ***MMSA-1*** mRNA and protein levels in ^***TCF4−/+***^U266 cells 1: U266 cells; 2: ^*TCF4−*^U266-NC; 3. ^*TCF4−*^U266 cells; 4: ^*TCF4+*^U266-NC; 5. ^*TCF4+*^U266 cells

We successfully constructed ^*TCF4****−/+***^U266 and ^*MMSA−1****−/+***^U266 stable cell lines using lentivirus tranfection protocols, and had been identified by qRT-PCR (Fig. 1A and B). In ^*TCF4*−^U266 and ^*TCF4*+^U266 cells, *MMSA-1* expression level was greatly down and up regulated both in mRNA level and protein level, as shown in Fig. 1C and D, indicating the regulation of *TCF4* on *MMSA-1* gene expression in U266 cells.

### chIP and dual-luciferase report assay showed TCF4 could bind with *MMSA-1* promoter sequences and greatly up regulate its activity

**Fig. 2:**
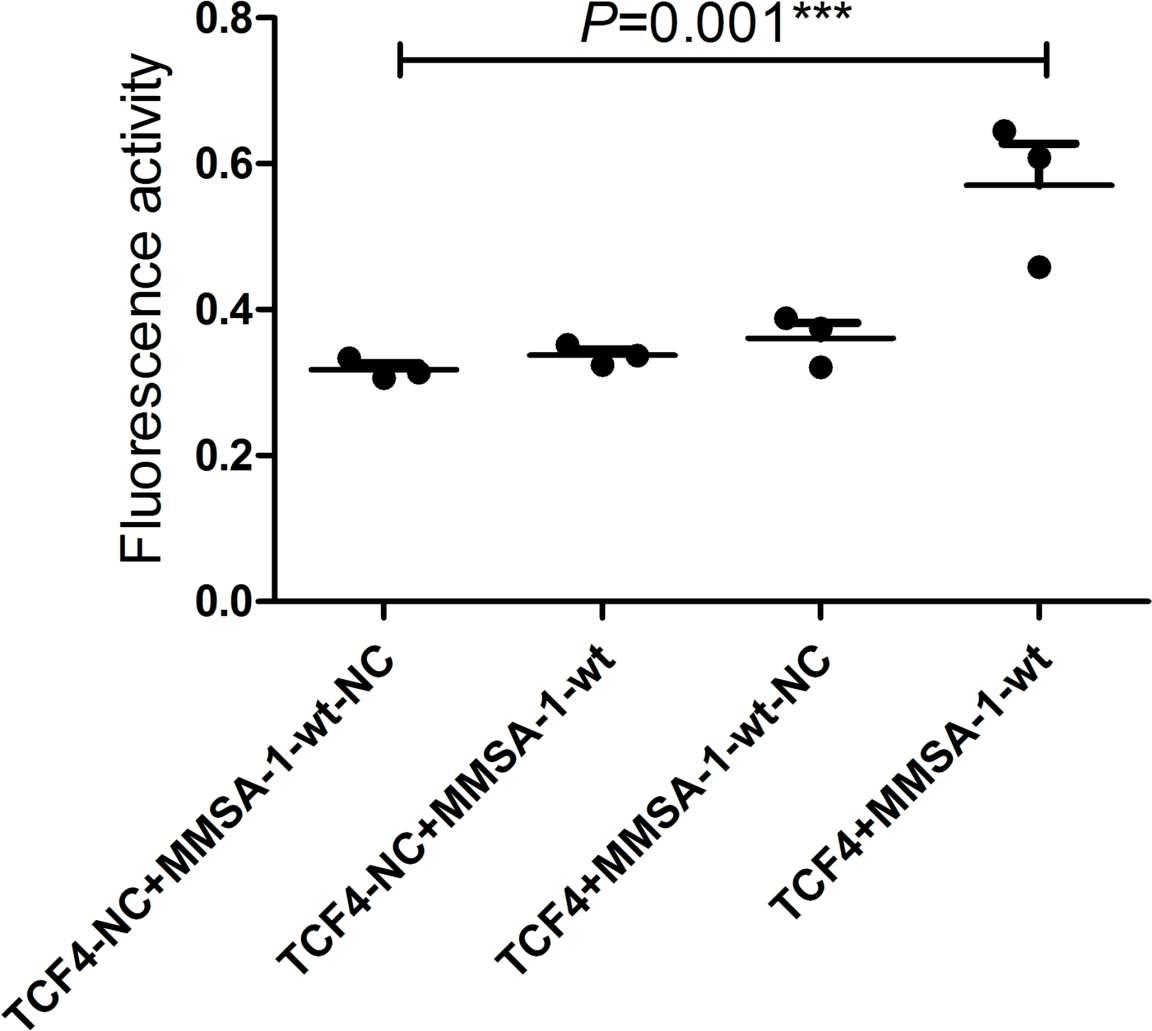
Dual-Luciferase Report Assay results in different U266 cells

Input *MMSA-1* primer sequences were designed and chosen, with primer 2 named *Homo-MMSA-1–2* of best amplification performance and ideal size. Its sequences were as follows: Forward: 5’- AGCCCCTTTCCTTTTTTG-3’, reverse: 5’- CACGTTGCCTGCTATTCC − 3’, with size of 254 bp. The result showed that in ^*TCF4+*^U266 cells, *Homo-MMSA-1–2* was much higher than that in the other U266 cells including U266 cells, ^*TCF4−*^U266, ^*TCF4−*^U266-NC and ^*TCF4+*^U266-NC, as shown in Supplementary Fig. 1. This result strongly proved the combination of TCF4 protein and *MMSA-1* promoter sequences in U266 cells. Figure 2 showed dual-luciferase report assay result, which suggested that the fluorescence activity in *TCF4 + MMSA-1*-wt group was much higher than that in the three control groups with *P* value of 0.001, indicating TCF4 could up regulate the promoter activity of *MMSA-1* gene.

### MMSA-1 and RAS proteins co-localization and interaction

**Fig. 3:**
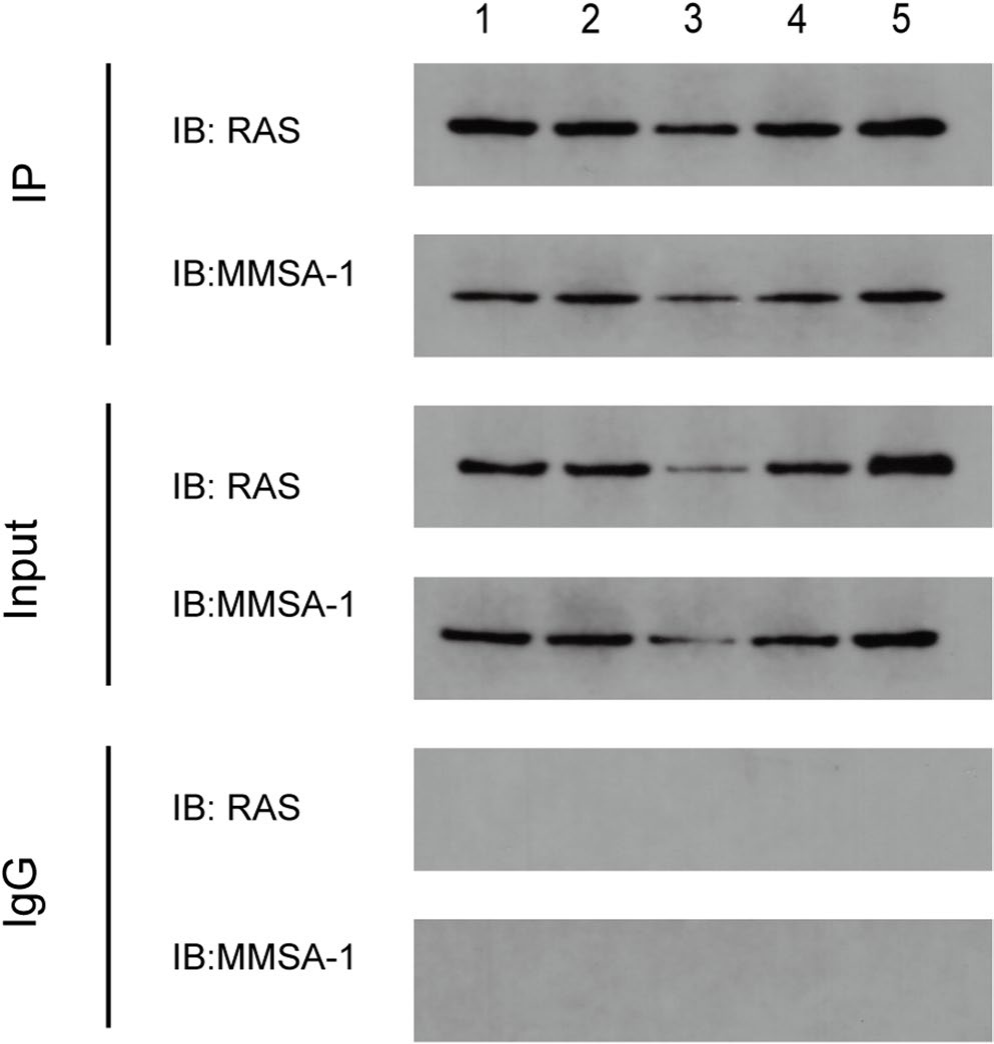
MMSA-1 and RAS co-localization and interaction in U266 cells 1: U266 cells; 2: ^*MMSA−1−*^U266-NC; 3. ^*MMSA−1−*^U266 cells; 4: ^*MMSA−1+*^U266-NC; 5. ^*MMSA−1+*^U266 cells

As shown in Supplementary Fig. 2, RAS protein (showed as green) was mainly expressed in cytoplasm while MMSA-1 (showed as red) was mainly localized on U266 cell membrane, with cell nucleus being stained with Dapi (showed as blue). We found that in ^*MMSA−1−*^U266 cells, both MMSA-1 and RAS protein expression level were much lower than that in U266 cells and in two control U266 cells while in ^*MMSA−1+*^U266 cells, MMSA-1 and RAS protein expression levels were much higher than the other U266 cells, indicating the up-regulation of MMSA-1 on RAS protein. Moreover, it seemed that higher expressed RAS was more localized on cell membrane. All these results suggested RAS protein expression level as well as cellular localization was regulated by MMSA-1 protein. And in Fig. 3, Co-IP result proved the interaction between MMSA-1 and RAS protein in U266 cells.

### MMSA-1’s activation on RAS downstream signaling pathways

**Fig. 4:**
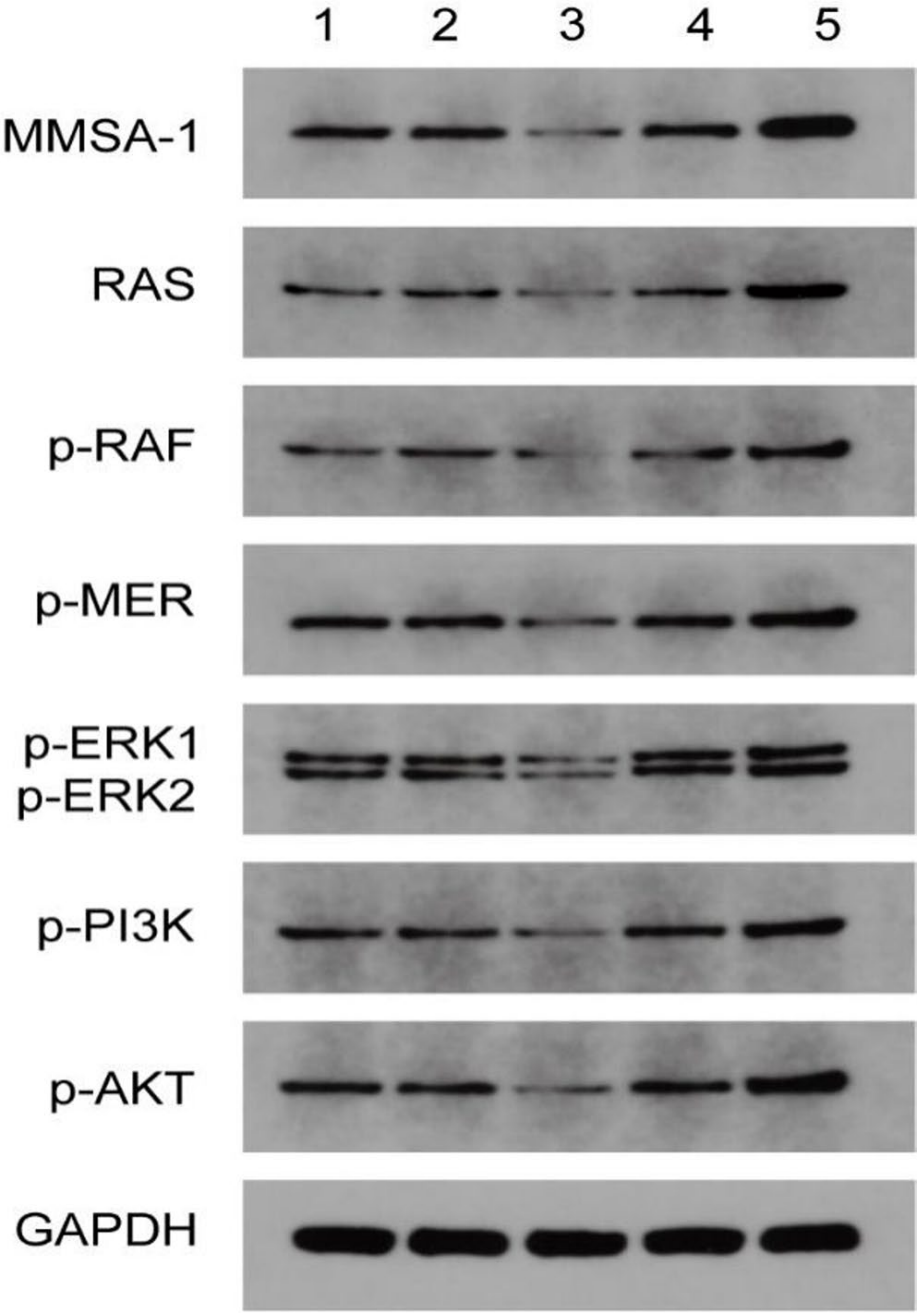
RAS downstream related molecule expressions in different U266 cells 1: U266; 2: ^*MMSA−1−*^U266-NC; 3. ^*MMSA−1−*^U266; 4: ^*MMSA−1+*^U266-NC; 5. ^*MMSA−1*+^U266

Western Blot was performed to analyze MMSA-1’s activation on RAS downstream signaling pathways including proliferation pathway *RAS/RAF/MEK/ERK* and anti-apoptosis pathway *RAS/RAF/PI3K/AKT*. The result showed that in ^*MMSA−1+*^U266 cells, both proliferation related molecules including p-RAF, p-MEK, p-ERK1/ERK2 and anti-apoptosis related molecules such as p-PI3K and p-AKT were significantly up regulated than in U266 cells as well as in the other controls cells, as shown in Fig. 4, suggesting the activation of MMSA-1 on RAS/RAF signaling pathways.

### MMSA-1’s effect on myeloma cells biological behavior including proliferation, migration and apoptosis

**Fig. 5:**
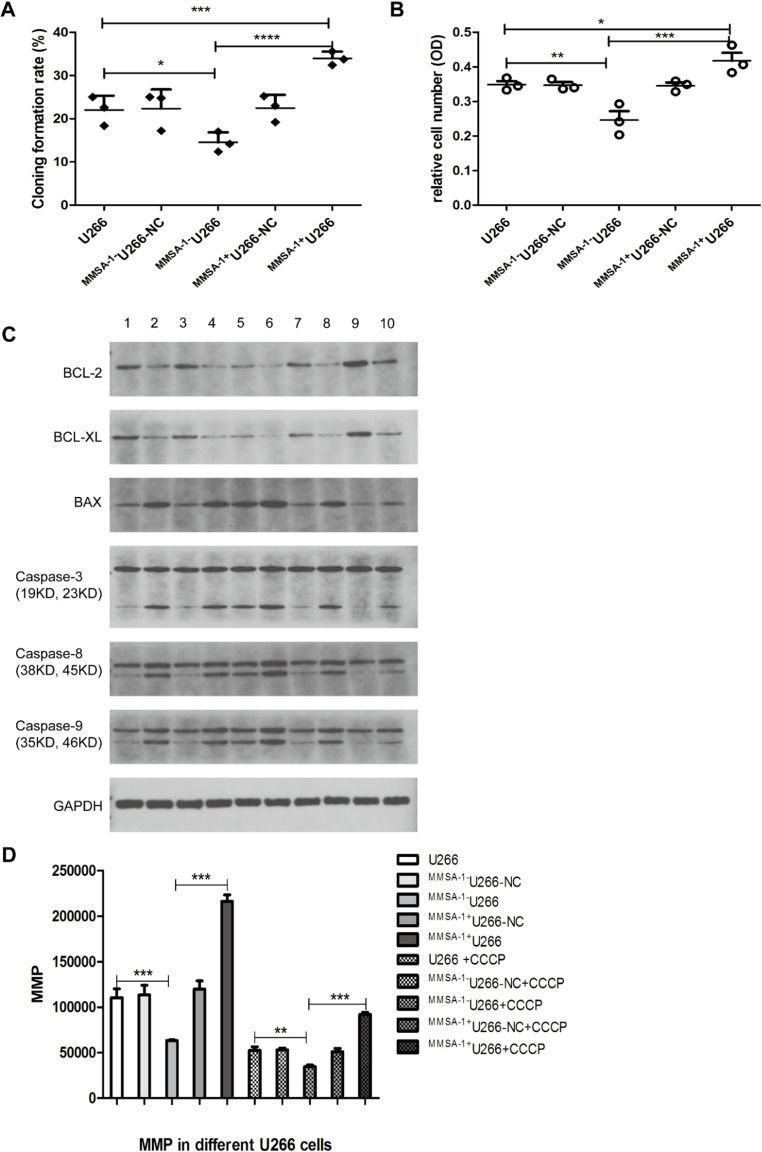
MMSA-1’s effect on U266 cells proliferation, migration and apoptosis** A** showed cloning formation assay result; **B** showed transwell assay result; **C** showed apoptosis related proteins expression and **D** showed mitochondrial apoptosis in different U266 cells. 1: U266; 2: U266 + CCCP-1; 3. ^*MMSA−1−*^U266-NC; 4. ^*MMSA−1−*^U266-NC + CCCP-1; 5. ^*MMSA−1−*^U266; 6: ^*MMSA−1−*^U266 + CCCP-1; 7. ^*MMSA−1+*^U266-NC; 8. ^*MMSA−1+*^U266-NC + CCCP-1; 9. ^*MMSA−1*+^U266; 10. ^*MMSA−1*+^U266 + CCCP-1

A series studies were performed to detect MMSA-1’s effect on myeloma cells biological behavior including clonogenecity capacity, migration ability and apoptosis. The result showed that in ^*MMSA−1+*^U266 cells, cloning formation rate was much higher than that in the other control group cells (Fig. 5A), so as the migration ability in transwell assay (Fig. 5B). As shown in Fig. 5C, the detected anti-apoptosis proteins BCL-2, BCL-XL level in ^*MMSA−1+*^U266 cells were much higher than that in the other U266 cells while apoptosis inducing protein BAX was much lower in ^*MMSA−1+*^U266 cells than that in the other cells, so as the great down regulation of Caspase family members in ^*MMSA−1+*^U266 cells, all of which suggesting the MMSA-1’s proliferation promoting and anti-apoptosis ability in myeloma cells.

Moreover, mitochondrial apoptosis in different U266 cells was also investigated. mitochondrial apoptosis was indicated by MMP. As shown in Fig. 5D, compared with U266 and ^*MMSA−1−*^U266, MMP in ^*MMSA−1+*^U266 cells was much higher than that in the other U266 cells, and when all of the cells were pretreated with CCCP, an inducer of cell apoptosis, the result was similar, all of which indicating the anti-apoptosis potential of MMSA-1 on U266 cells.

### MMSA-1’s effect on the interaction between myeloma cells and bone marrow microenvironment

**Fig. 6:**
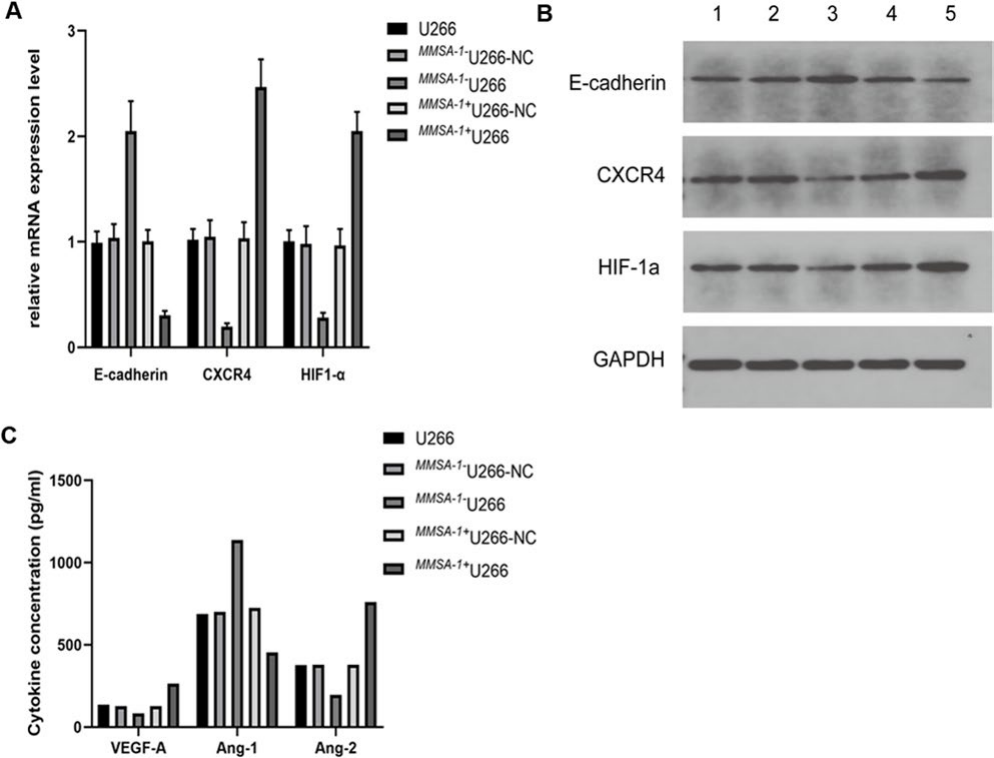
Adhesion molecules and angiogenesis factors levels in different U266 cells** A** showed qRT-PCR result and **B** showed Western Blot result of adhesion molecules; **C** showed angiogenesis factors levels. 1: U266 cells, 2: ^*MMSA−1−*^U266-NC; 3. ^*MMSA−1−*^U266; 4. ^*MMSA−1+*^U266-NC; 5. ^*MMSA−1+*^U266

In this part, MMSA-1’s effect on the interaction between myeloma cells and bone marrow microenvironment was also investigated. We focused on the hypoxic microenvironment, adhesion molecules expression and angiogenesis factors in the microenvironment. Our results showed that in ^*MMSA-1+*^U266 cells, hypoxia-inducible factor HIF-1a and adhesion molecule CXCR4 was significantly up regulated, while E-cadherin was greatly down regulated, compared with the other U266 cells, as shown in Fig. 6A and B. Moreover, we also found that angiogenesis promoting factors such as VEGF-A, Ang-2 were dramatically elevated, while Ang-1 was significantly regulated, as shown in Fig. 6C. All these results proved the pivotal role of MMSA-1 played in the interaction between myeloma cells and bone marrow microenvironment. By inducing hypoxia environment, reducing adhesion molecules and promoting angiogenesis in the bone marrow, MMSA-1 ultimately reduced the interaction between myeloma cells and bone marrow environment, helped myeloma cells and disease progression.

## Discussion

Our previous studies have proved that *MMSA-1* was highly and specifically expressed on both myeloma cell lines as well as primary myeloma cells from MM patients, which high expression was closely related with patients’ disease status and was also an independent prognostic factor for myeloma patients [4]. As a very important signaling pathway, *Wnt/β-catenin/TCF4* plays a pivotal role in kinds of human malignancies such as breat cancer, hepatocarcinoma, endometrial carcinoma, colorectal cancer as well as MM [10, 11]. Using bioinformatic tools we found something interesting that in *MMSA-1* promotor sequence, there may be binding site of transcription factor *TCF4*, which means *MMSA-1* gene might be regulated by *Wnt/β-catenin*/*TCF4* signaling pathway in myeloma cells. Herein in this study we firstly constructed TCF4 down/up regulated U266 stable cell lines successfully, termed as ^*TCF4−*^U266 and ^*TC4+*^U266 cells, respectively. Furthermore, we used dual-luciferase report assay and chIP assay to explore TCF4’s binding and regulation on *MMSA-1* promoter activity. The result showed that TCF4 could indeed bind with *MMSA-1* promoter sequences and dramatically up regulate its activity, which induced abnormal hyperactivation of *MMSA-1* promoter and in the end resulted in the over expression of MMSA-1 protein in U266 cells.

Orthologues of *RAS* were originally discovered in cancer-causing viruses in rats, leading to the identification of three endogenous human *RAS* genes, *NRAS*,* KRAS*, and *HRAS* [12, 13]. It has been proved that these small GTPases could activate RAF kinases, which in turn phosphorylate MEK and finally ERK-kinases, and in the end involved in cells proliferation, growth, adhesion and apoptosis and play essential roles in human malignancies. In MM pathogenesis, *RAS/RAF/MERK/ERK*-pathway mutations account for most of the pathway overactivation while there are still some hyperactivation cannot be explained by gene mutation [14]. That’s to say, some other overactivation mechanism exists in myeloma cells [15, 16]. In our study, we found that *RAS* was over activated by *MMSA-1*, both in mRNA level and protein levels, just like its overactivation mechanism by *ZDHHC9* in breast cancer [6]. Furthermore, we also found that in U266 cells, MMSA-1 and RAS protein were mainly co-localized on U266 cells membrane, with little expressed in U266 cells cytoplasm, as shown by Immunofluorescence assay. And the proteins interaction was also highly enhanced after MMSA-1 expression was elevated, as shown by Co-IP result. These findings strongly proved MMSA-1’s regulation and impact on RAS protein’s biological function.

What happened after RAS was over activated by *MMSA-1* in myeloma cells? In order to find out RAS related downstream signaling pathways changes after MMSA-1 expression was elevated, including proliferation pathway *RAF/MEK/ERK* as well as anti-apoptosis pathway *RAF/PI3K/AKT*, we performed a series of assays and found that hyperactivation of RAS led to phosphorylation of kinds of kinases including RAF, MERK, ERK-1 and ERK2, which in the end promoting myeloma cells proliferation. So as PI3K and AKT, which were also phosphorylated by RAS, and both of which were significantly over activated, which resulted in inhibition of myeloma cells apoptosis and prolonged their survival.

Hyperactivation of *MMSA-1/RAS/RAF* signaling pathway made great changes of myeloma cells biological behaviors, including the stronger ability to proliferate, longer survival time, greater invasion and migration capabilities, as shown by the clonogenicity forming assay, Western Blot for key apoptosis factors, and mitochondrial apoptosis result as well as transwell assay results. All these findings indicated the great impact of MMSA-1 expression level on myeloma cells survival. The precise molecular mechanisms by which MMSA-1 regulates the expression of BCL-2, BCL-XL, and BAX, including whether it involves transcriptional or post-transcriptional regulation, remain an important scientific question for future investigation.

It has been known that the interaction between myeloma cells and bone marrow microenvironment plays crucial supportive roles in myeloma cells differentiation, proliferation, survival, and drug resistance of the malignant plasma cells [17, 18]. The interaction could activate and amplify the upstream signals such as proliferation, anti-apoptosis to help myeloma cells survival, proliferation and metastasis, which also closely related with extramedullary disease (EMD), an aggressive form of MM characterized by the ability of a clone and/or subclone to thrive and grow independent of the bone marrow microenvironment [19, 20]. What we were quite interested was whether MMSA-1 could also affect the interaction between myeloma cells and bone marrow microenvironment [21].

Adhesion molecules, being considered as lifeline of multiple myeloma cells, play essential part in the composite network of interaction [22]. The levels of expression and activity of these adhesion molecules are controlled by cytoplasmic operating mechanisms, as well as by extracellular factors including enzymes, growth factors and microenvironmental conditions. As an important biomarker, E-cadherin participated in the process of metastasis of MM cells through Epithelial-to-mesenchymal transition-like features as solid tumor [23]. And it was also believed to activate tumor-promoting properties in plasmacytoid dendritic cells [24]. Negative E-cadherin expression on BM myeloma cell membranes was also proved to associated with extramedullary disease [25]. In our study, the result showed that MMSA-1 could significantly inhibit E-cadherin level, which provided support for myeloma cells dissemination.

CXCR4 is a pleiotropic chemokine receptor that is expressed in approximately 60% of primary MM cells from the BM [26]. By binding with its ligand, stromal cell-derived factor 1α (SDF-1α), the SDF-1α/CXCR4 axis activates a variety of intracellular signaling pathways that modulates those biological processes that related with proliferation, survival, invasion, dissemination, metastasis, and drug resistance [27]. It has been reported that A significant decrease in SDF-1α plasma levels and CXCR4 expression on MM cells in the apheresis product compared with those in BM before mobilization was observed [28]. In this study, we demonstrated that after MMSA-1 expression was significantly inhibited, CXCR4 expression was also greatly reduced, which indicating the migration promoting ability of *MMSA-1* on myeloma cells.

Angiogenesis is another important marker of myeloma cell survival [29]. Bone marrow angiogenesis in MM is regulated by angiogenic cytokines such as VEGF, fibroblast growth factor-basic (bFGF), Ang-1, Ang-2 and the degree of bone marrow angiogenesis has an impact on disease progression in MM [30, 31]. Of all the angiogenic cytokines, VEGF is the most widely studied cytokine in human malignancies. Multiple investigations have demonstrated that VEGF is significantly and directly correlated with micro vessel density in patients with multiple myeloma [32], which, in turn, is significantly correlated with patients’ overall survival as well as progression free survival [33, 34]. As a critical member of the angiopoietin family, Ang-1 plays essential role in orchestrating blood vessel formation in concert with VEGF. It could bind specifically to Tie-2 receptor (tyrosine kinase receptor with immunoglobulin and epidermal growth factor homology domain 2) which presents on the endothelial cells and helps in vessel stabilization and maturation. Numerous studies have proved that circulating Ang-1 level was reduced in newly diagnosed, symptomatic myeloma patients, resulting in an induced ratio of Ang-1/Ang-2, and which had been proved to correlate with adverse disease features, including International Staging System (ISS)-stage, extensive bone disease and renal impairment in newly diagnosed MM [35]. In our study, we also found that over expressed MMSA-1 could greatly increase VEGF-A level, reduce Ang-1 level and to reduce adhesion molecules expression including E- cadherin, CXCR4 and improve myeloma cells clonogenicity and migration ability, just as reported previously [36].

Hypoxia inducible factor-1 alpha (HIF-1α) was another proangiogenic molecule which was thought to be induced by both microenvironmental hypoxia and genetic mutations such as Lysine Demethylase 3 A and hexokinase-2 [37–39]. The elevated expression of HIF-1α has been proved to promote cellular processes and facilitate tumor progression [40]. It has also been reported to be increased in MM and correlated significantly with serum β2-microglobulin levels and increases from stage I to III [41], and related with an inferior progression survival and overall survival in myeloma patients [42]. In our study we also found that MMSA-1 could greatly elevated HIF-1α levels secreted by U266 cells compared with the control group cells, indicting the MMSA-1 impact on myeloma hypoxia-induced processes.

## Conclusions

To conclude, we revealed for the first-time myeloma specific antigen *MMSA-1’*s molecular regulation mechanism in MM as well as its biological function in promoting myeloma cells proliferation, apoptosis and its dissemination and invasion course, which greatly deepened our understanding of the biological function of *MMSA-1* gene and provide a novel treatment target potential in the future. Of Course, the mechanistic data in this study are primarily based on the U266 cell line. While the data are internally consistent and compelling, future validation in other multiple myeloma cell lines, such as RPMI-8226 and NCI-H929, will be a crucial step to confirm the broader applicability of ***MMSA-1***’s functions.

## Supplementary Information

Below is the link to the electronic supplementary material.

Supplementary Material 1

Supplementary Material 2

## Data Availability

The datasets supporting the conclusions of this article are all included within the article. Other datasets are available from the corresponding author on reasonable request.

## Abbreviations

MM: multiple myeloma
MMSA-1: Multiple Myeloma Specific Antigen-1
TCF4: transcription factor 4
CXCR4: C-X-C chemokine receptor type 4
STR: short tandem repeat
GAPDH: glyceraldehyde-phosphate dehydrogenase
EV: empty vector
qRT-PCR: quantitative reverse transcription polymerase chain reaction
ChIP: Chromatin immunoprecipitation
PCR: polymerase chain reaction
HRP: horseradish peroxidase
PBS: phosphate buffered solution
FITC: fluorescein isothiocyanate
Dapi: 4’,6-Diamidino-2’-phenylindole
Co-IP: Co-immunoprecipitation
BCL-2: B cell lymphoma-2
BCL-XL: B cell lymphoma- extra large
ELISA: enzyme-linked immunosorbent assay
BAX: BCL-2 associated X protein
MMP: mitochondrial membrane potential
VEGF: vascular endothelial growth factor
EMD: extramedullary disease
SDF-1α: stromal cell-derived factor 1α
bFGF: fibroblast growth factor-basic
ISS: international staging system
HIF-1α: Hypoxia inducible factor-1 alpha
