# Supplementary material for: MMSA-1 is regulated by Wnt/TCF4 and involved in multiple myeloma progression and invasion via RAS/RAF signaling pathway
Source: Ann Hematol. 2026 Jan 15;105(1):11. doi: 10.1007/s00277-026-06740-8 (PMC12804270; doi:10.1007/s00277-026-06740-8)

Article title: *MMSA-1* is regulated by *Wnt /TCF4* and involved in multiple myeloma progression and invasion via *RAS/RAF* signaling pathway

Journal name: Annals of hematology

Author names: Shan Meng*, Hailing Liu, Liufang Gu, Jin Wang, Jianli Wang, Wanhong Zhao

Affiliations: Department of Hematology, the Second Affiliated Hospital of Xi'an Jiaotong University, 710004, Xi'an, Shaanxi Province, P.R. China

Correspondence: Shan Meng, Department of Hematology, the Second Affiliated Hospital of Xi'an Jiaotong University, West Five Road, NO. 157, Xi'an, Shaanxi Province, P. R. China; Phone: 86-29-8767-9457, Fax: 86-29-8767-8634, Email: 101xyz2@163.com

Supplementary data 1:

The promoter sequence of *MMSA-1* gene

https://www.ncbi.nlm.nih.gov/nuccore/NC_000023.11?report=genbank&from=129803288&to=129843886&strand=true)

ORIGIN

1 ccatttggcc agtggtgggc ggttgccaca gctggtttag ggccccgacc actggggccc

61 cttgtcagga ggagacagcc tcccggcccg gggaggacaa gtcgctgcca cctttggctg

121 ccgacgtgat tccctgggac ggtccgtttc ctgccgtcag ctgccggccg agttgggtct

181 ccgtggttca ggtaacgagg ggtgggggcg ggggtctcct gggctcggct gcagggtggg

241 ctgcctcccc ttccgcggcg cctggaggcg gggacgtcgg caccgccctt ctcaactcgg

301 cagccgccga gcccctggcc cggggtgact acacgtggcg gggcgtgtgt gagtgtgtac

361 gagcgtgtgg ctgcgtgtgc gtgtggatgt ggcgcgcgtc ccaacctgcc tggggcggtg

421 gtccgcggcg tggcggagtc agggcgggcc tcgagaggcc gcgcccgcgg ctcggccccg

481 gactccggcc tccggcctcc ggcccccccgc tggggagccg gtcgctgctt cccgacggat

541 tttgatcttt gttcttccag gccggctccc ccttcctggt ctcccttctc ccgctgggcc

601 ggtttatcgg gaggagattg tcttccaggt tagttttaca caaaggacgg gttctctgca

661 cctctgggcc cccctcccct tccccgaacc ttctaccttc tcgtttccct gctccgctcc

721 taggctgttt ttggaccttt gccctctcca gcccgcgagt tggctgtttc tccctccgtc

781 cgtcgggctg caggcttccg cctcagtcct gccagcgtgg ggcatgtggt gtcggtaaat

841 agaggccaca acaccacacc tgagcggagg agaagggtag cagaggctga gaaactctgg

901 gcctgggaat tcaggatgct gagtagagat ggtacttagg acatgtccca gaaagcgccc

961 ttgtgacttt tcttagctgc tgccattccc caccccccaa ccagccatca gcagtcacca

1021 accattccag aatctctcct gcttaagtca gcctttcacc tacaggtctc tgatgtggtt

1081 tcattaagat tctcacaaat gttaggctag accagccaga ttgtttcatt tcccggactc

1141 aggaagttgc agagggtttg atgaggggaa atctgttttg tgtaaataca atgcacgtat

1201 gtgcacagag cgtccagaac ctcctggccc tgccagaggg tgacttgttc cctgctgtga

1261 ttcatattcg agtggctaat tgaagaaaga aggtgaagtg gaggaaggga gggaggtttt

1321 ataaggacag aaactctact attattttta gagtcctttt aatgatgttg aaagaacact

1381 gtggcatagt ggaacatgcc cttgactgga agttagcagt atcagcattg ctgctgtgtg

1441 gtcctggata agttatttcc tctctctggt cctcagttta ctcatttaca aaacaaagta

1501 ttaataattg gcacgatcac ctataaaatc ctctctagtt ccaaggttct gtgatgatca

1561 gatcatcagc attagattgg aagcatagag gcttatttaa gaagaaatac tagccccttt

1621 cctttttga gaacctttta tcataccttc ttttagagaa caaacacgaa ttggaaataa

1681 acttttccca cttccccgtc cccacccctt cagtcagttt tcaactttct gtctctggga

1741 aaggagggtt gaactgtatt ttcttattat tgcctcattt ttttttcttt ttctgctttt

1801 tcttagggct agcaattgga cttttgatga tgtttgaccc agcggcagga atagcaggca

1861 acgtgatttc aaagctgggc tcagcctctg tttcttctct cgtgtaatcg caaaacccat

1921 tttggagcag gaattccaat ca

Supplementary Fig. 1 chIP results in different U266 cells In **A**(a-e), 1 indicated DNA lander, 2 indicated Input, 3 indicated TCF4 antibody, 4 and 5 indicated negative and positive control, respectively. **A**(f) showed agarose gel electrophoresis result of the broken DNA fragments, 1 indicated DNA lander, 2-6 indicated different U266 cells.

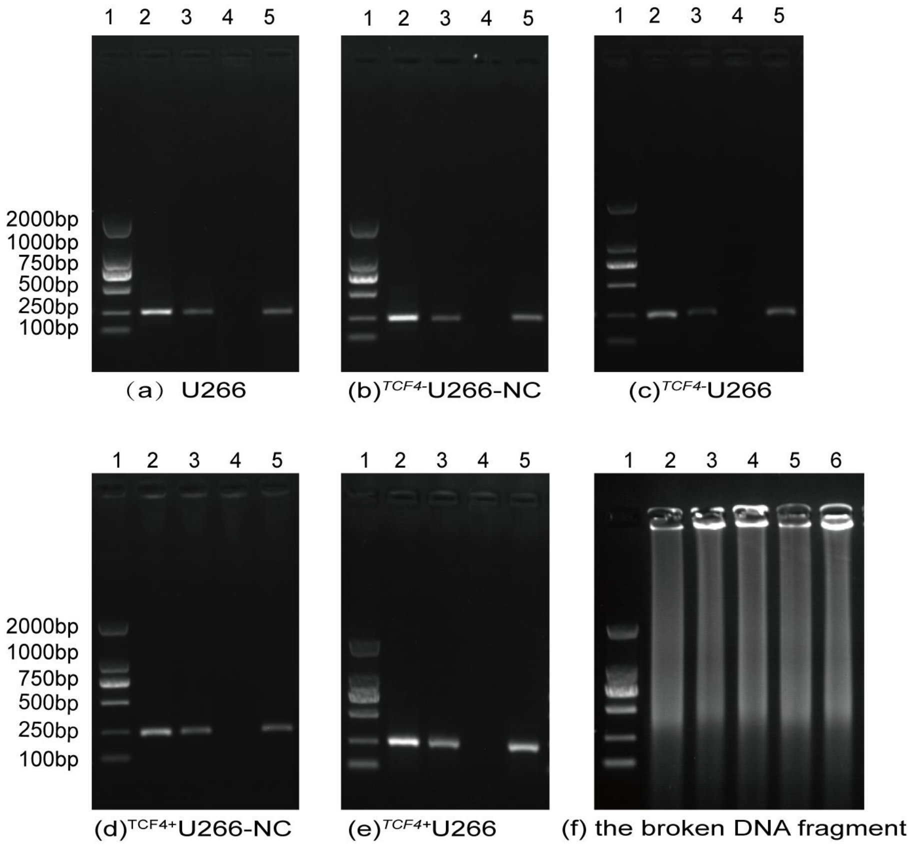

Supplementary Fig. 2 MMSA-1 and RAS co-localization RAS protein showed as green and MMSA-1 showed as red, with cell nucleus being stained with Dapi (showed as blue)

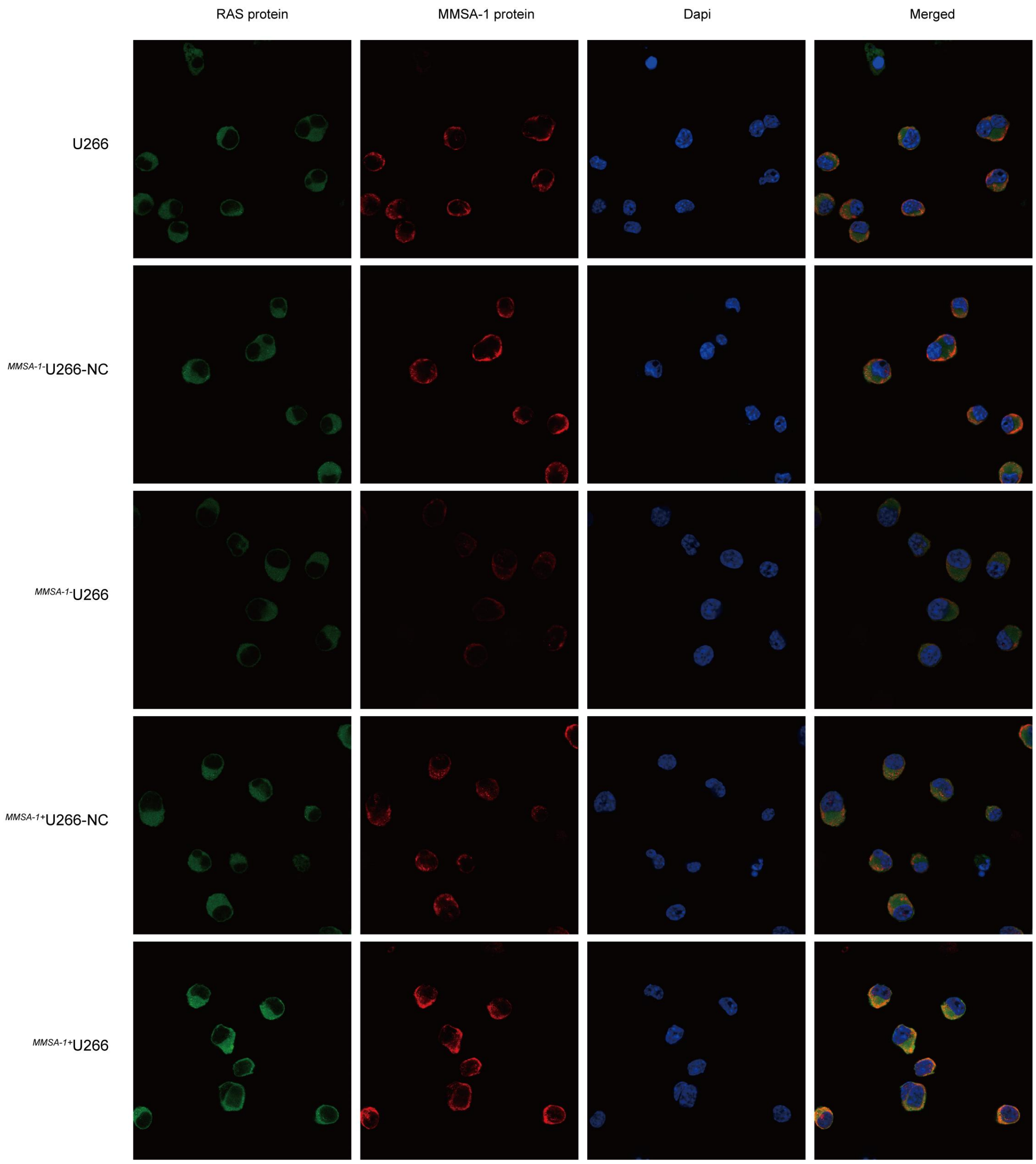

Supplement: Supplementary file 1 — Supplementary Material 1 [file 277_2026_6740_MOESM1_ESM.pdf]
