# Supplementary figures and images for: MMSA-1 is regulated by Wnt/TCF4 and involved in multiple myeloma progression and invasion via RAS/RAF signaling pathway
Source: Ann Hematol. 2026 Jan 15;105(1):11. doi: 10.1007/s00277-026-06740-8 (PMC12804270; doi:10.1007/s00277-026-06740-8)

Fig 1D

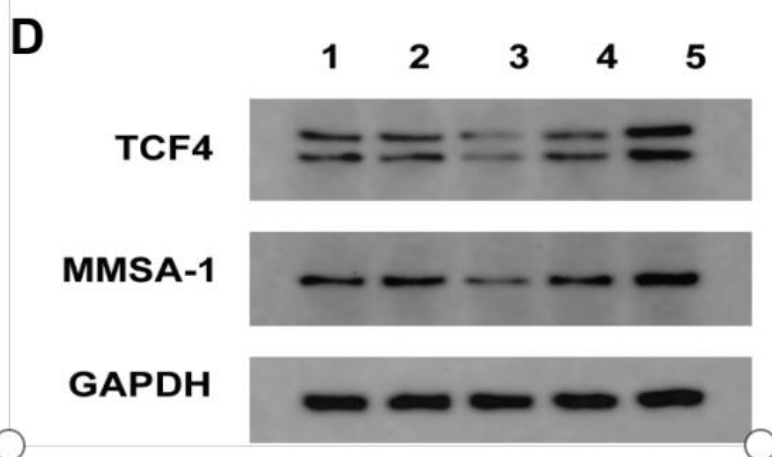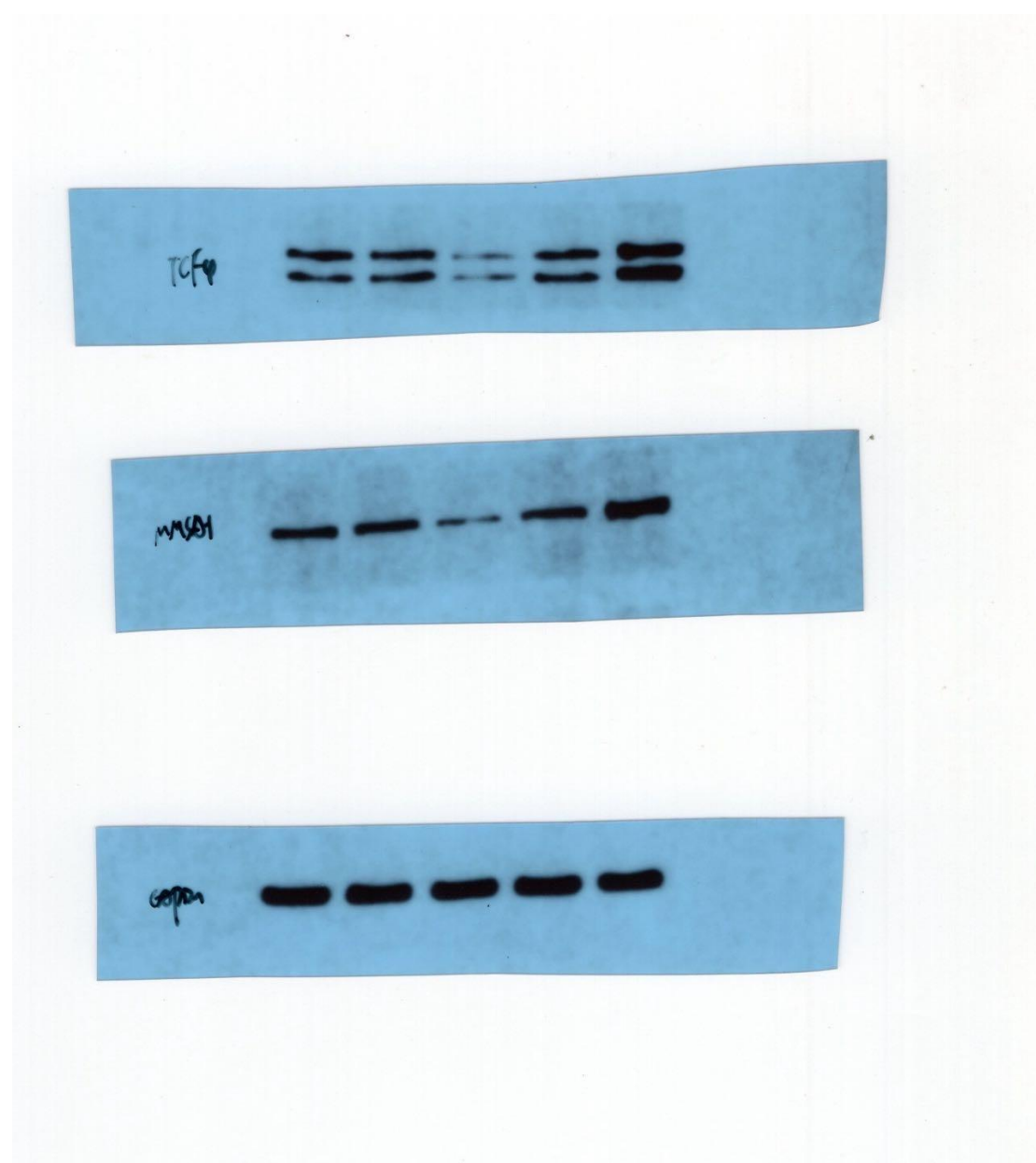

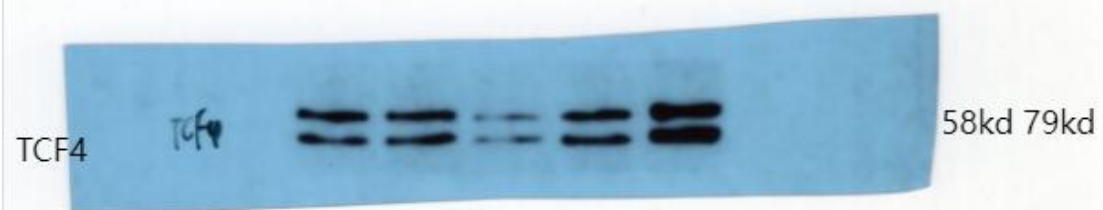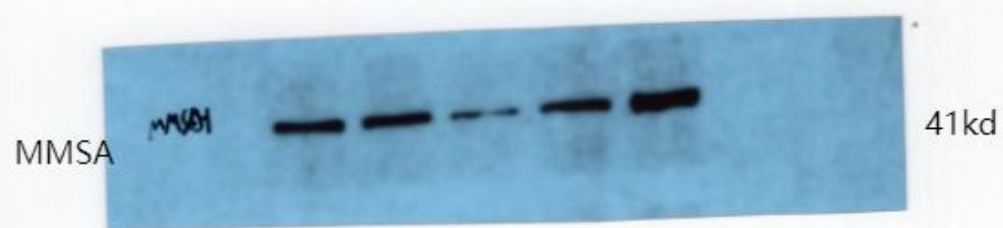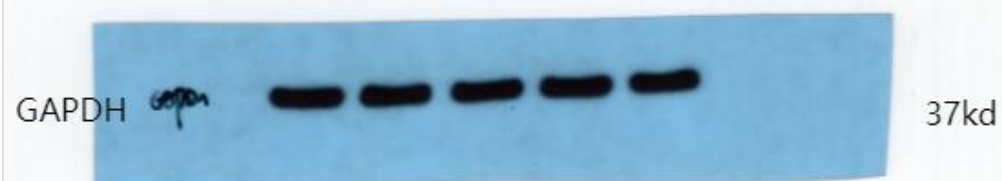

Fig 3B

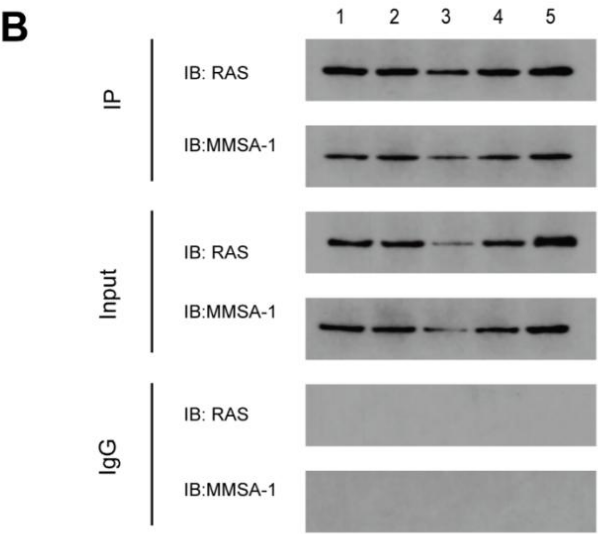

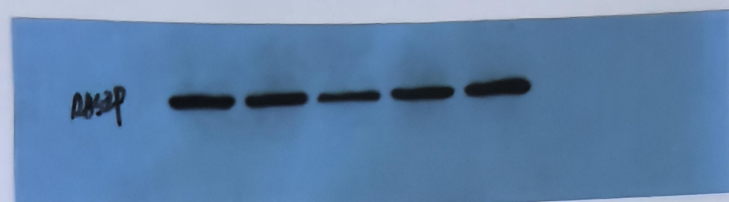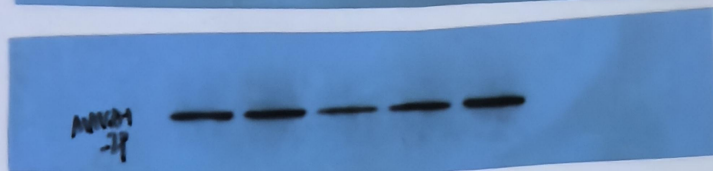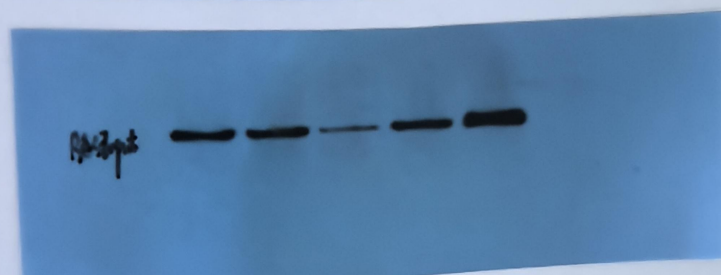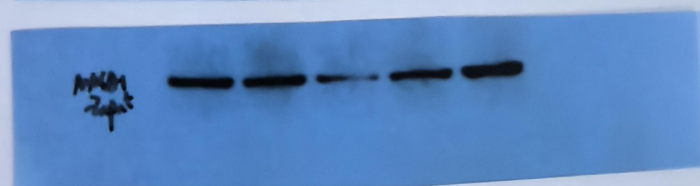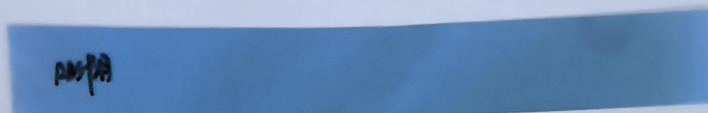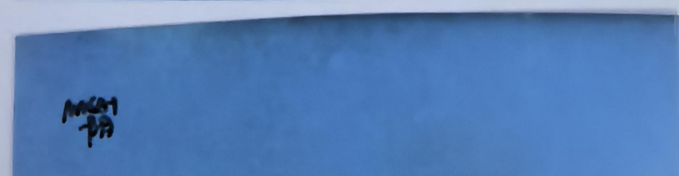

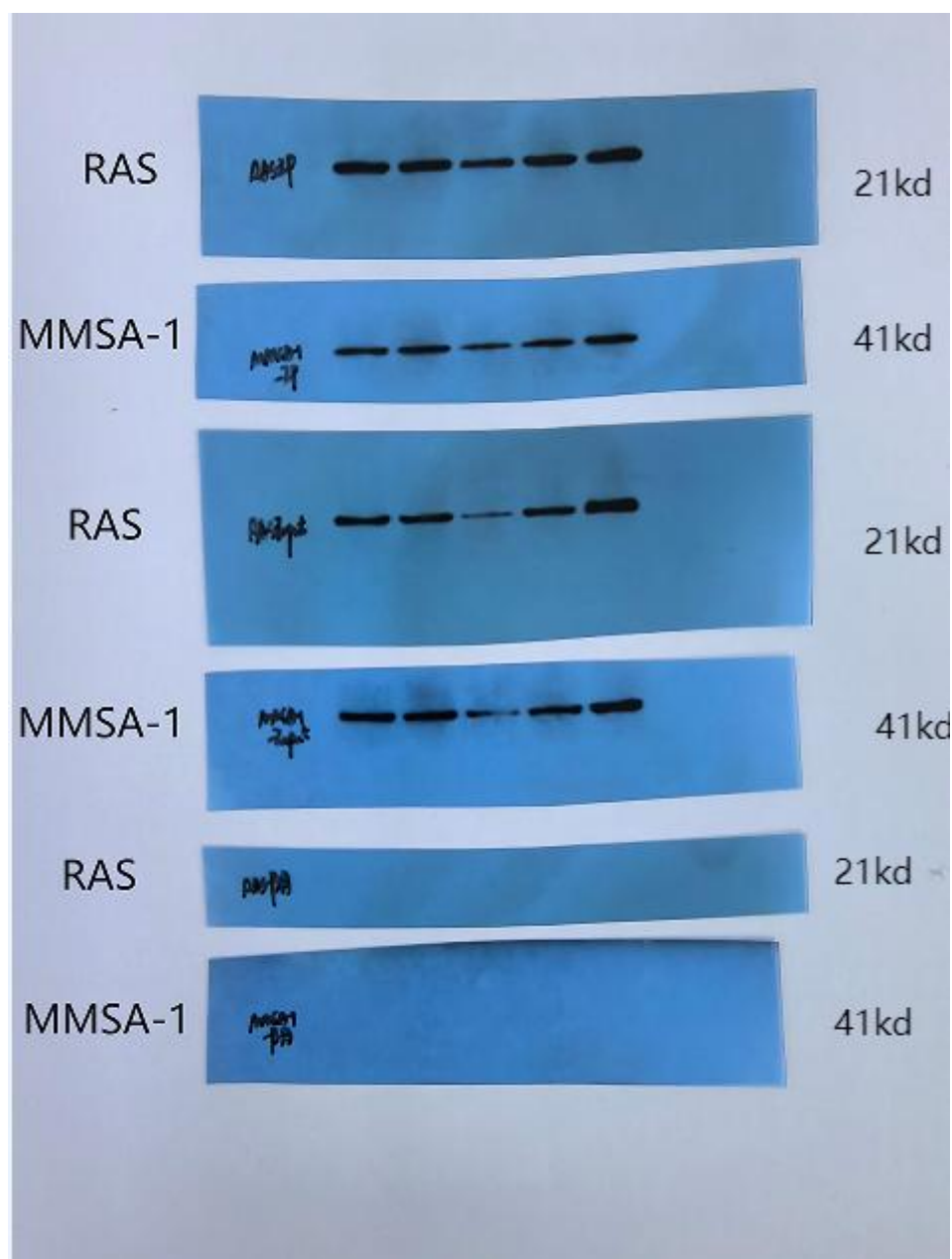

Fig 4

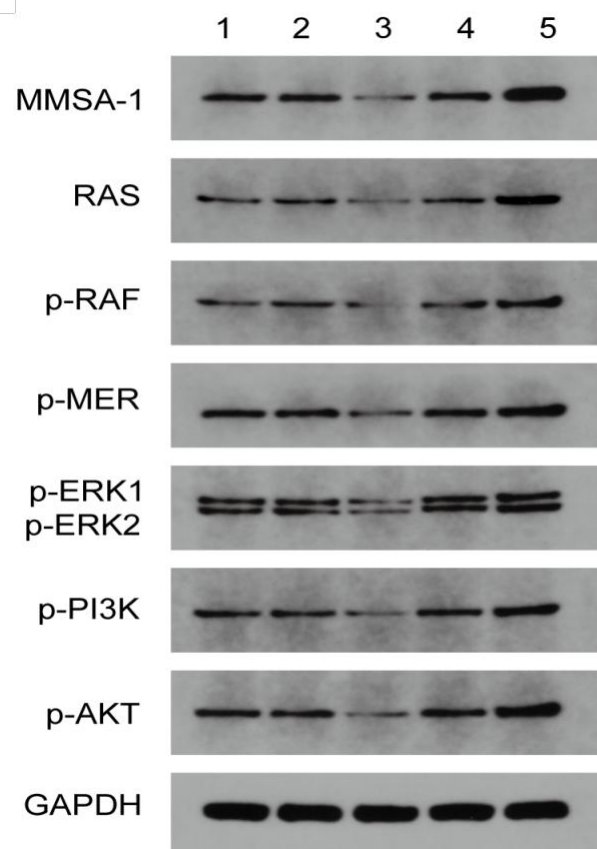

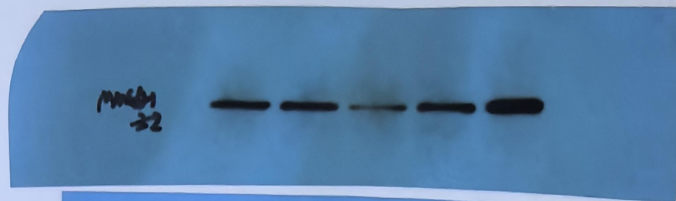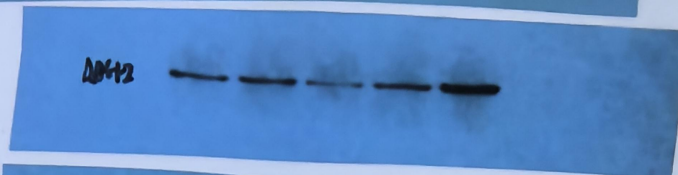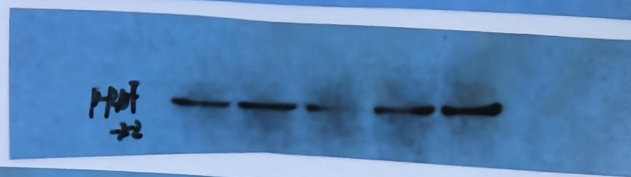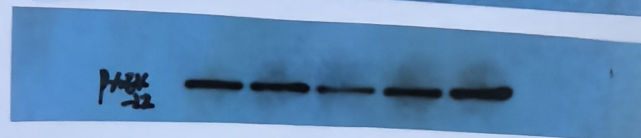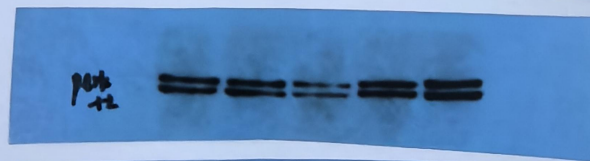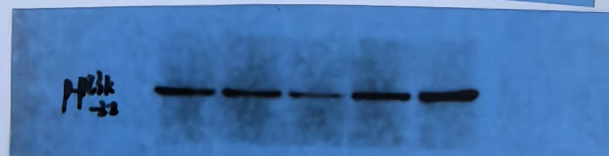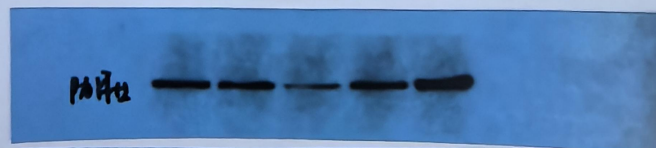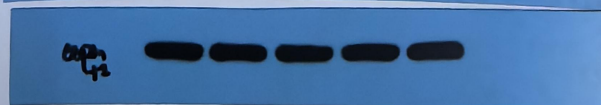

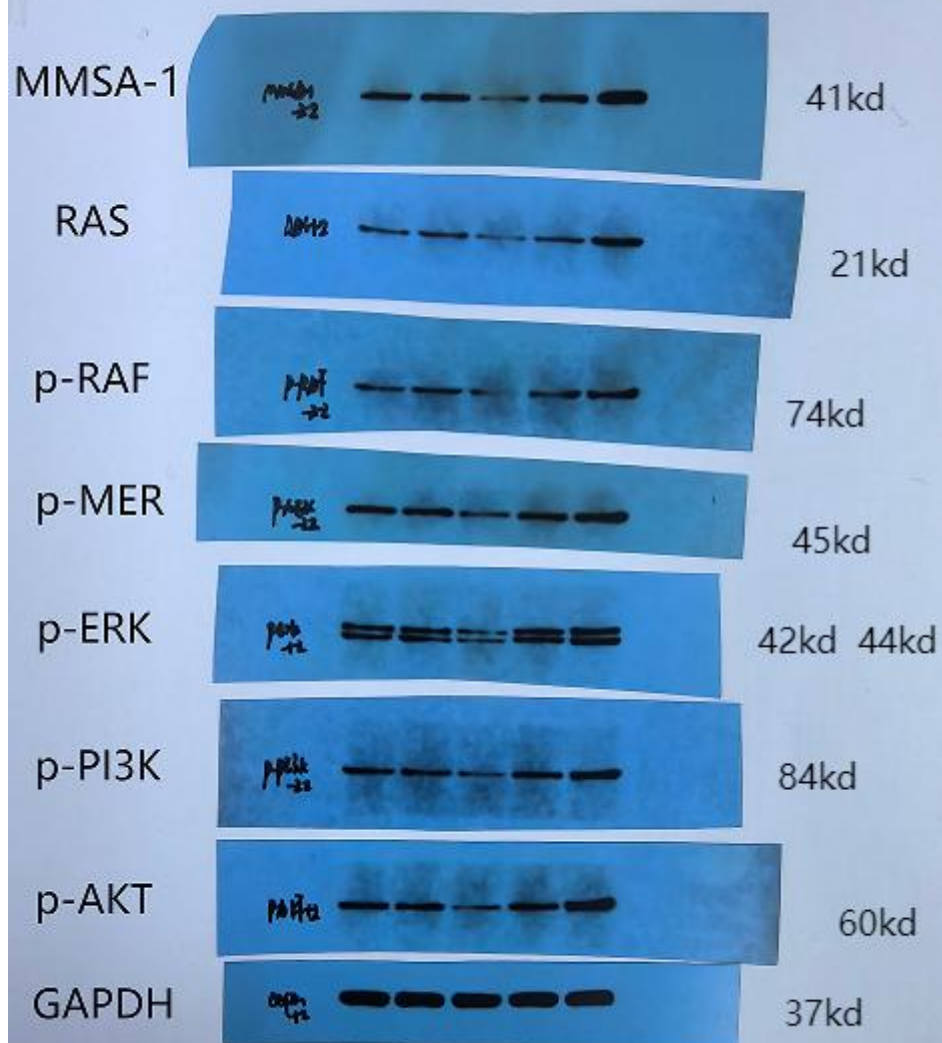

Fig 5C

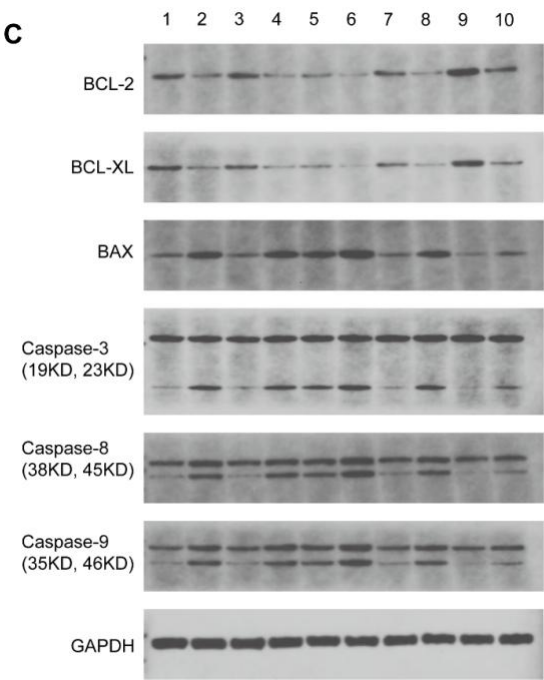

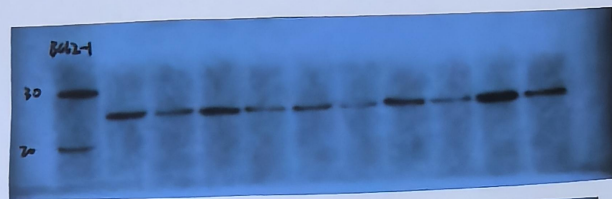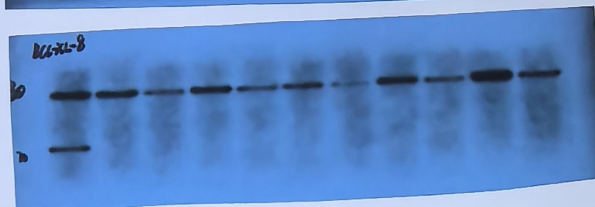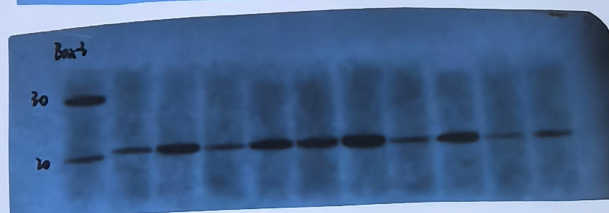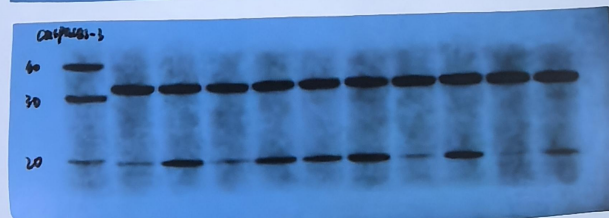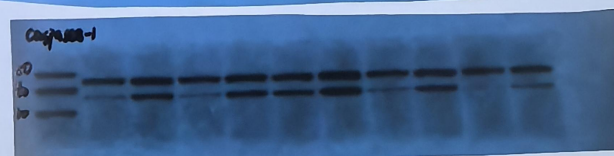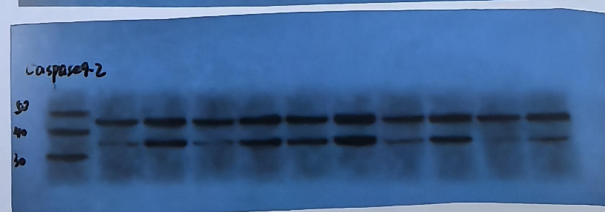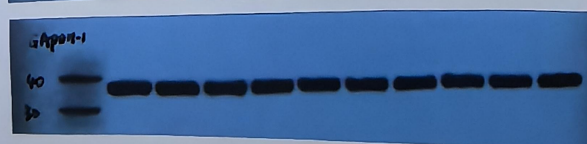

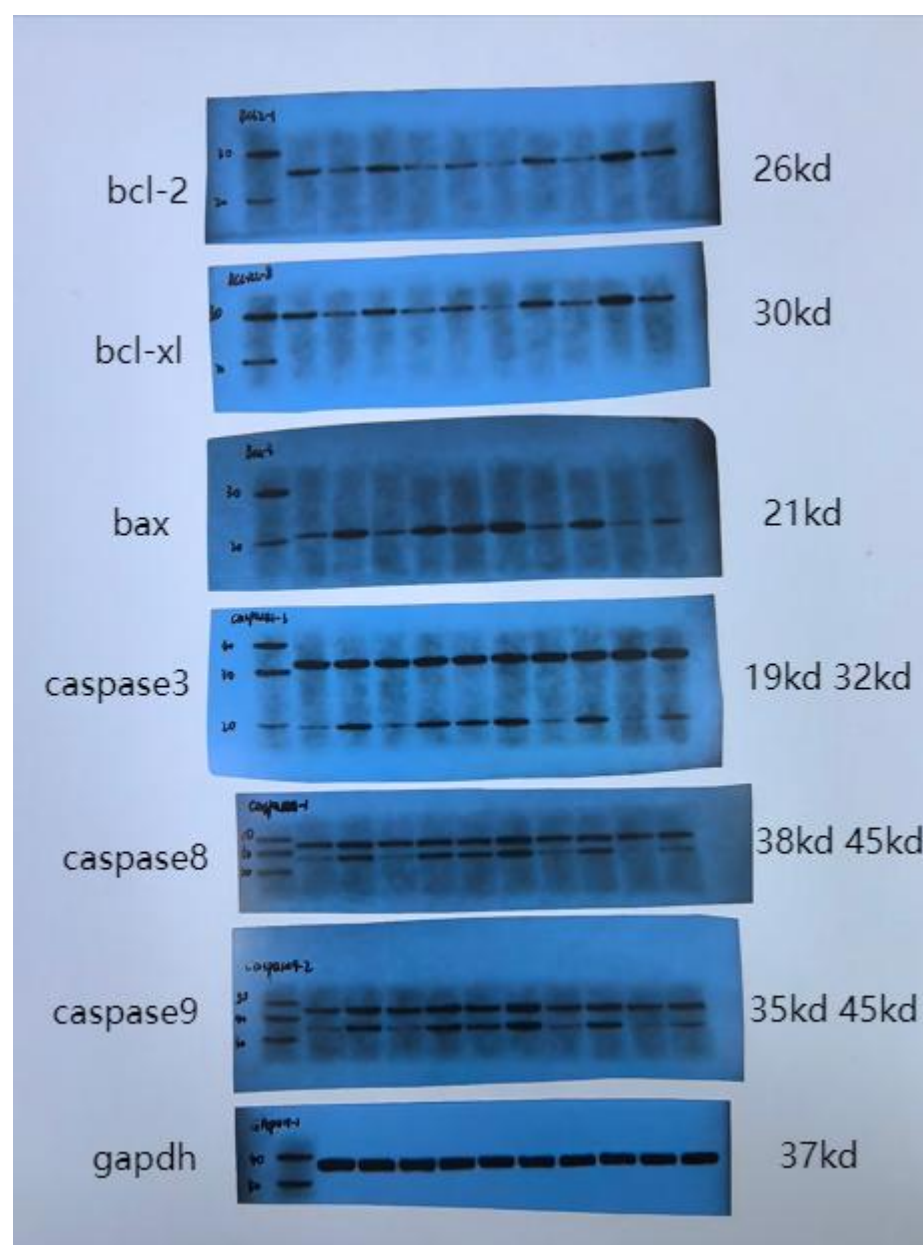

Fig 6

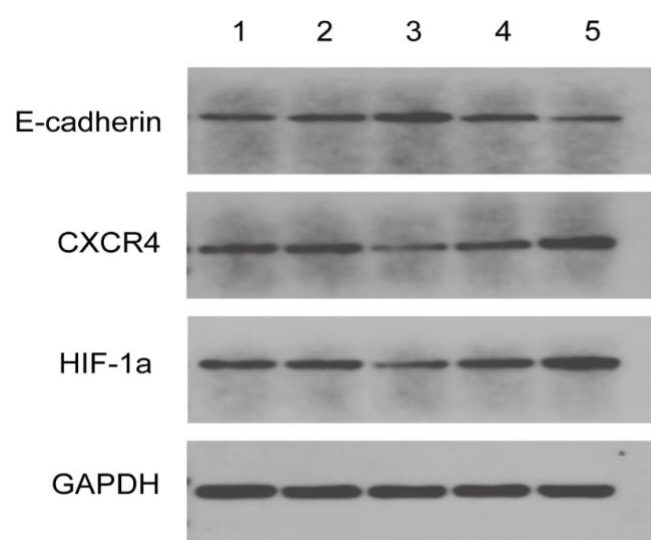

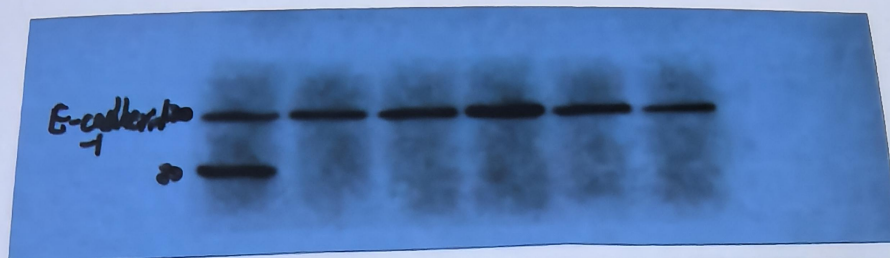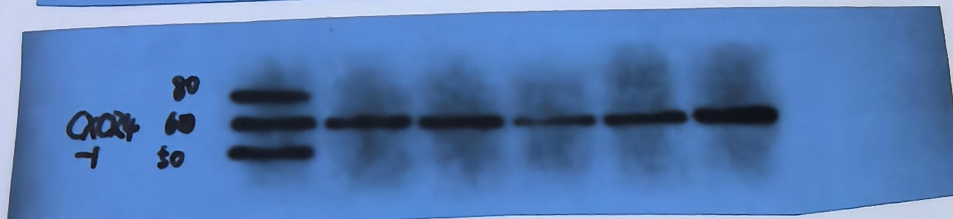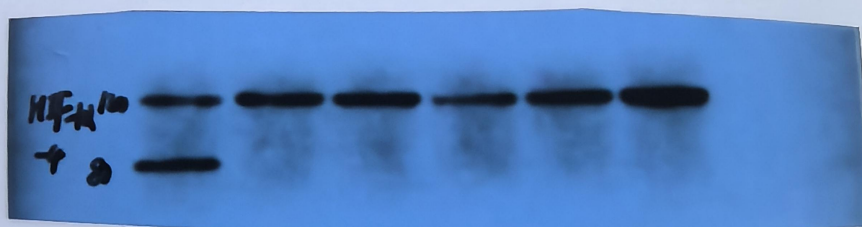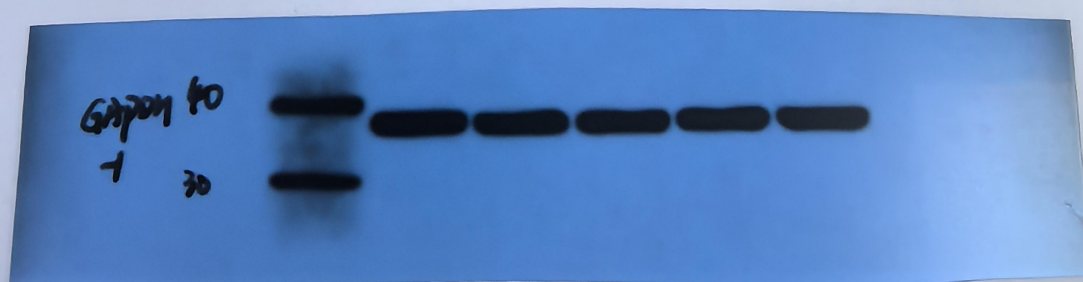

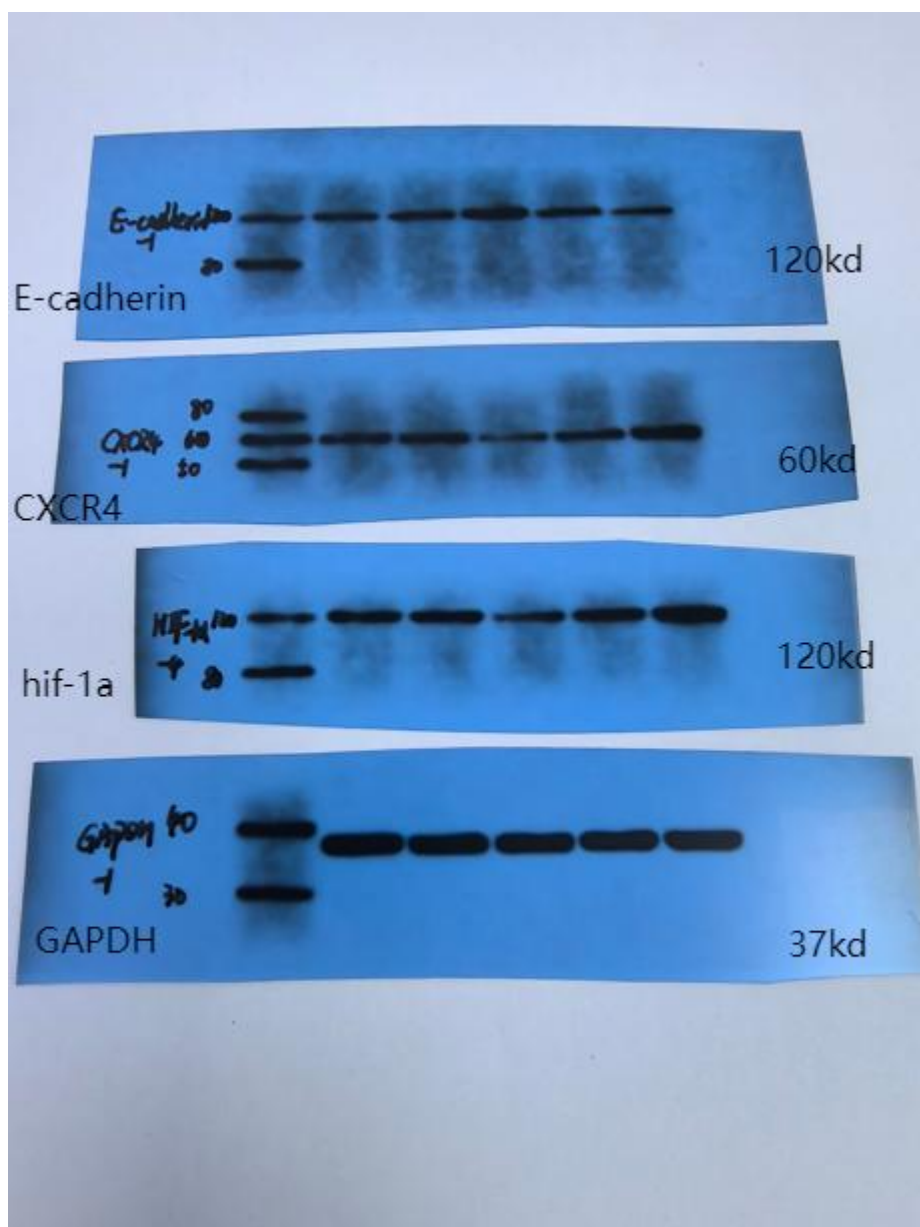

Supplement: Supplementary file 2 — Supplementary Material 2 [file 277_2026_6740_MOESM2_ESM.pdf]
